# A Readiness Level Assessment of Healthcare Facilities in the Democratic Republic of Congo for the Management of Cardiovascular Disease and Diabetes

**DOI:** 10.3390/jcm14103498

**Published:** 2025-05-16

**Authors:** Karl B. Angendu, Francis K. Kabasubabo, Julien Magne, Pierre Z. Akilimali

**Affiliations:** 1 Inserm U1094, IRD UMR270, CHU Limoges, EpiMaCT—Epidemiology of Chronic Diseases in Tropical Zone, Institute of Epidemiology and Tropical Neurology, OmegaHealth, University of Limoges, 87000 Limoges, France; karl.angendu_baki@unilim.fr (K.B.A.); francis.kabasubabo@etu.unilim.fr (F.K.K.); julien.magne@unilim.fr (J.M.); 2 The Democratic Republic of Congo National Public Health Institute, Kinshasa P.O. Box 3243, Congo; 3 Faculty of Medicine, Christian University of Kinshasa, Kinshasa P.O. Box 834, Congo; 4 Patrick Kayembe Research Center, Kinshasa School of Public Health, University of Kinshasa, Kinshasa P.O. Box 11850, Congo; 5 Department of Nutrition, Kinshasa School of Public Health, University of Kinshasa, Kinshasa P.O. Box 11850, Congo

**Keywords:** non-communicable diseases, cardiovascular diseases, diabetes, readiness assessment, service availability

## Abstract

**Introduction**: Sub-Saharan Africa in general, and the Democratic Republic of the Congo (DRC) in particular, is undergoing an epidemiological transition characterized by a more rapid increase in the number of non-communicable diseases (NCDs). However, the level of readiness of the DRC’s healthcare facilities (HFs) to manage these diseases is unknown. Thus, our study aimed to assess these HFs’ level of readiness to manage cardiovascular disease (CVD) and diabetes. **Methodology**: This cross-sectional study involved 1412 HFs in the DRC, selected by stratified random sampling. They are representative of the country’s 26 provinces. The World Health Organization (WHO) Service Availability and Readiness Survey (SARA) was used. The “readiness” outcome was a composite measure of the capacity of HFs to manage CVD and diabetes. The readiness indicator comprised four domains, and a score of ≥70% indicated “readiness” to manage CVD and diabetes. Informed consent was obtained from the stakeholders, and the ethics committee held a positive opinion. Statistical analyses were performed using STATA 17 software. **Results**: The average readiness scores of the DRC’s HFs to manage CVD and diabetes are less than 50%, being 38.3% (37.3–39.3) and 39.8% (38.7–40.9), respectively. These scores were less than 40% for CVD and diabetes in rural HFs. They were less than 30% for CVD and diabetes in primary-level HF. No province possesses over 50% of health facilities equipped to address cardiovascular illnesses, and only four provinces (Haut Uele, Kinshasa, Nord Kivu, and Sud Kivu) possess over 50% of health facilities equipped to address diabetes. The provinces with health facilities exhibiting the least preparedness in managing cardiovascular illnesses and diabetes are Nord Ubangi and Sankuru. Only 0.07% (0.01–0.5) of HFs obtained a score ≥ 70% for CVD management, and 5.9% (4.8–7.3) obtained this score for diabetes management. **Conclusions**: Significant deficiencies must be rectified to enhance service delivery in the management of cardiovascular disease (CVD) and diabetes. Most primary-level and rural facilities demonstrated inadequate preparedness for CVD and diabetes screening and management, exhibiting low readiness scores and limited-service availability in the assessed domains. While secondary-level services are relatively accessible, critical gaps persist that must be addressed to improve readiness for CVD and diabetes care. Healthcare facilities should possess the capacity to deliver recommended services across various tiers, ensuring both service readiness and availability.

## 1. Introduction

Sub-Saharan Africa is undergoing an epidemiological transition characterized by a more rapid increase in the number of non-communicable diseases (NCDs) than of communicable diseases (CDs) [1]. This trend is also observed for mortality, where NCDs have overtaken CDs as the leading cause of death in many low-income countries [1,2]. This evolution is the result of changing lifestyles, notably rapid urbanization, sedentary lifestyles, and the Westernization of lifestyles [3,4,5]. This new epidemic is fueled by a decline in physical activity, changes in diet, and improved life expectancy at birth [2,6].

Wilbroad Mutale and colleagues reported that research conducted jointly by the World Economic Forum and Harvard University showed that NCDs could cost the global economy USD 47,000 billion over the next few years, equivalent to 75% of global gross domestic product and a cost greater than that of the global financial crisis [7]. They also reported that this amount is much higher than the estimated USD 11.4 billion per year needed by low- and middle-income countries to implement effective strategies for the prevention and treatment of NCDs [7]. Epidemiology in the Democratic Republic of the Congo (DRC) remains essentially focused on CDs despite the epidemiological transition seen in tropical areas [1]. The latest data from studies on NCDs in the DRC, particularly diabetes and hypertension (HTN), show that NCDs represent a major public health problem [8,9]. Paradoxically, given the current epidemiological trend of NCDs in the DRC, the level of readiness of healthcare facilities (HFs) to manage these diseases is not known.

Two studies attempted to ascertain the level of preparedness of HFs for managing diabetes and HTN, both conducted in Kinshasa with a methodological approach that differs from that recommended by the World Health Organization (WHO) [10,11]. In the two prior studies, the suggested and standard domains and associated tracers were not used. The level of preparation was not assessed using a standardized model employing scoring that creates clear judgment criteria. Accurate information on the preparedness of health services is essential for identifying deficiencies to enhance healthcare quality in the DRC. The World Health Organization (WHO), in conjunction with the United States Agency for International Development (USAID), established a methodology and suite of tools known as the Service Availability and Readiness Assessment (SARA) to furnish stakeholders with insights into the healthcare system’s performance over time. The Service Provision Assessment (SPA) is a health facility survey that gathers data on service availability and Quality of Care (QoC) metrics within a nation’s health system. Our evaluation of the SPA, concentrating on cardiovascular disease (CVD) and diabetes healthcare services, may prove valuable in pinpointing gaps and opportunities to fortify primary healthcare (PHC) within the DRC health system. Nonetheless, no comprehensive national studies have evaluated the preparedness of healthcare facilities to address diabetes and CVD using standardized methodologies. Consequently, our study sought to fill this gap by evaluating the preparedness of HFs in the DRC to manage cardiovascular disease and diabetes using standard tools and approaches.

## 2. Methodology

### 2.1. Design and Sampling

This study was based on a cross-sectional survey of health service providers. Randomly selected HFs (public, private, and denominational) at all levels of the health pyramid in the country’s 26 Provincial Health Divisions (PHDs) were included. Data collection took place from 16 October 2017 to 20 April 2018. The current study is part of a larger survey entitled “Evaluation of Health Care Services” [12]. A total of 1412 HFs were surveyed using the WHO Service Availability and Readiness Survey (SARA) [13], following the example of other studies carried out in countries in the same context as the DRC [7,14,15,16,17].

Based on the HF database provided by the National Health Information System (NHIS), 12,050 HFs were identified, listed, and used as the sampling frame for the study. These were public, private, and denominational HFs comprising health centers (HCs), reference health centers (RHCs), referral general hospitals (RGHs), hospital centers (HCs), and clinics. Using the sampling frame provided by NHIS, the HFs were selected by stratified random sampling according to province, first taking into account the explicit type of HF and then the implicit type of membership, with an average of 50 HFs per province [12].

The sample was executed at the national level, considering the weight of each province in the health facility survey database (sampling frame) of the DRC. Consequently, Bas-Uele had the fewest HFs, while North Kivu had the most. The exact number of HFs was distributed according to the weight of the province, ensuring that all health districts (HDs) were considered. The weight was adjusted for non-response and then standardized [12].

### 2.2. Data Collection and Quality Assessment

The validated WHO SARA was used to collect data for this study. Each HF was assessed based on four domains (Table 1): (1) staff and guidelines, (2) technology and basic equipment, (3) diagnostic capacity, and (4) essential medicines. Information on these four domains for an HF was mainly gathered from the head of the HF or a member of the management team with sufficient knowledge of the hospital’s capacity and operation.

In cases where the HF manager could not be reached, the next person in line was contacted. Where such information was available, the investigators verified its existence to supplement and assess the quality of the information provided by the health authority.

Two hundred and eighty interviewers were recruited and trained to collect data using the tablets and the paper version of the tool. This made it possible to set up 70 four-person teams, including three interviewers and a supervisor. All had a degree in human medicine or nursing. Details of the training and methodological approach used during the survey are provided in the survey report [12].

Quality control of electronically transmitted data was carried out at two levels: (i) in the field by supervisors, before sending the data to the server managed by the Kinshasa School of Public Health (KSPH), and (ii) at the KSPH. Paper questionnaires were also sent to the KSPH and entered manually. For each HF, the data transmitted from the field and those entered at the KSPH were compared. In this way, harmonizations were made, and a single file of audited data was created.

### 2.3. Readiness Assessment and Data Analysis

The standard WHO SARA, an HF assessment tool, was used to collect the data required for the assessment [13]. Based on the WHO SARA guidelines, the questionnaire included 13 assessment tracers for diabetes management and 12 tracers for cardiovascular disease (CVD), divided into four domains (Table 1): (i) personnel and guidelines, (ii) basic technologies and equipment, (iii) diagnostics, and (iv) essential medicines. The tracers for these four areas were compiled to calculate the readiness score.

Questionnaires completed in the software were meticulously checked to reduce errors. The study outcome variable, “readiness”, was assessed for 1412 HFs providing diabetes and CVD care services across the country’s 26 PHDs. The “readiness” outcome was a composite measure of an HF’s ability to manage diabetes and CVD. The readiness indicator comprised the 4 domains mentioned above, and each domain consisted of a set of tracer elements. Service readiness was assessed in four stages: (i) determining the availability of diabetes care services and CVD readiness indicators at each HF level; (ii) calculating tracer element index scores (number of tracer elements present × 100/number of tracer elements that should be present); (iii) calculating the HF Readiness Index (RI) according to the 4 domains (the average of all the tracer element index scores in each domain); and (iv) calculating the average readiness score (RS) at the HF level (the average of the RI of the 4 domains). Scores were stratified by province and compared with a threshold score of 70%. This threshold was chosen on the basis of studies conducted using the SARA tool, considering an HF to be “ready” to manage diabetes and CVD if it achieved a score of at least 70% [7,18,19,20]. All data were analyzed using STATA version 17.

### 2.4. Statistical Methods

The collected data were transferred to Stata version 17 for data quality assessment before statistical analysis began. Variables were categorized according to WHO recommendations and the DRC context. HFs were grouped into “primary level” and “secondary level”. The primary level comprised HCs and RHCs. The secondary level comprised hospitals, RGHs, HCs and clinics. They were also categorized according to their administrative anchorage or direct administrative authority: “public”, “private/NGO” and “denominational”. Finally, they were organized according to the urbanization of their geographical location: “rural area” and “urban area”. Categorical variables were reported as frequencies (%). Associated confidence intervals were also reported. Quantitative variables were reported as mean (standard deviation) and median [interquartile range]. Domain scores were estimated by calculating the ratio of available tracers to required tracers. The mean of the domain scores was used to calculate the average HF score. The score at the provincial and national level was estimated by calculating the average HF score. The preparation level was calculated on the basis of the initial threshold. As the CVD and diabetes readiness score variables do not follow a normal distribution, the Mann–Whitney U test was used to compare the two means. When modalities were greater than 2, the Kruskal–Wallis test was performed. Normality was tested using the Shapiro–Wilk test. The significance level was set at 0.05. These calculations were carried out for CVD and diabetes. The results of the study were presented in the form of figures and tables using STATA 17 software, as well as Microsoft Office Excel 2024.

### 2.5. Ethical and Legal Aspects

To ensure confidentiality, we identified the variables that guarantee anonymity in the database. Informed consent was obtained from all participants in the survey. We had no contact with patients, and no biological procedures were used for data collection or processing. The results of this study will only be used in relation to its objectives, and no conflicts of interest are to be reported.

## 3. Results

**Figure 1:**
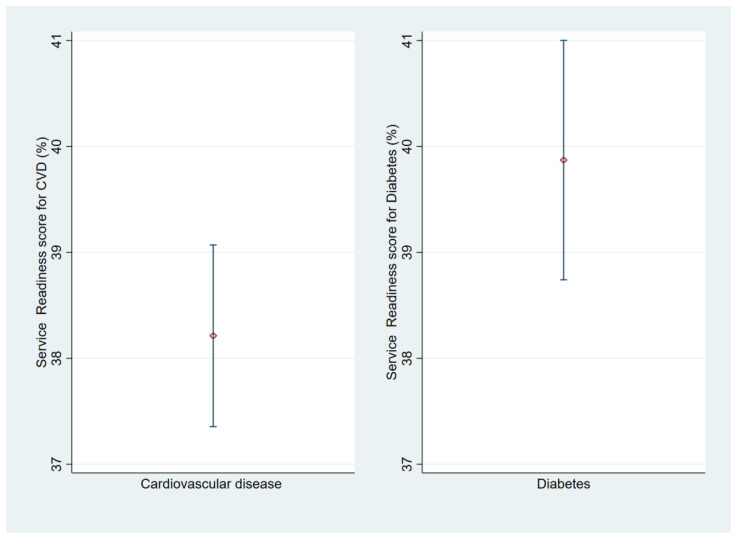
Average readiness scores of DRC HFs to manage CVD and diabetes.

**Figure 2:**
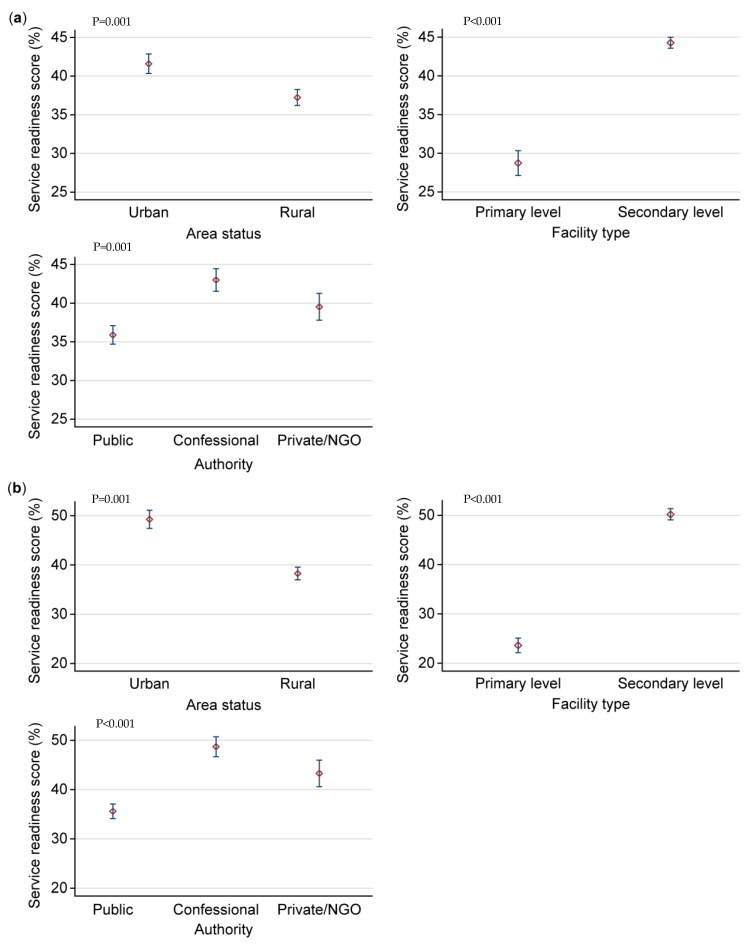
Average scores by stratification according to level of readiness of DRC HFs to manage CVD and diabetes. (**a**) Average scores stratified according to level of readiness of DRC HFs to manage CVD. (**b**) Average scores stratified according to level of readiness of DRC HFs to manage diabetes.

The average readiness scores in percentage of the DRC’s HFs able to manage CVD and diabetes were less than 50% for each of these diseases (Figure 1). They were, respectively, 38.3% (CI95%: 37.3–39.3) and 39.8% (CI95%: 38.7–40.9). The average stratification scores are shown in Figure 2. In connection with CVD (Figure 2a), urban HFs were significantly better prepared to manage CVD (41.7% (CI95%: 40.2–43.2)) than those in rural areas (*p* = 0.001). Secondary-level HFs were significantly more prepared to manage CVD (44.5% (CI95%: 44.0–45.0)) than primary-level HFs (*p* < 0.001), and confessional HFs were significantly more prepared to manage CVD (42.5% (CI95%: 41.8–43.3)) than public and private/Non-Governmental Organization (NGO) HFs (*p* = 0.001). As shown in Figure 2b, urban HFs were significantly more prepared to manage diabetes (49.0% (CI95%: 47.5–51.5)) than rural HFs (*p* = 0.001). Secondary-level HFs were significantly more prepared to manage diabetes (50.0% (CI95%: 48.5–51.5)) than primary-level HFs (*p* < 0.001), and denominational HFs were significantly more prepared to manage diabetes (48.5% (CI95%: 46.5–50.5)) than public and private/NGO HFs (*p* < 0.001).

### 3.1. Readiness of DRC HFs by Domains of Assessment

**Figure 3:**
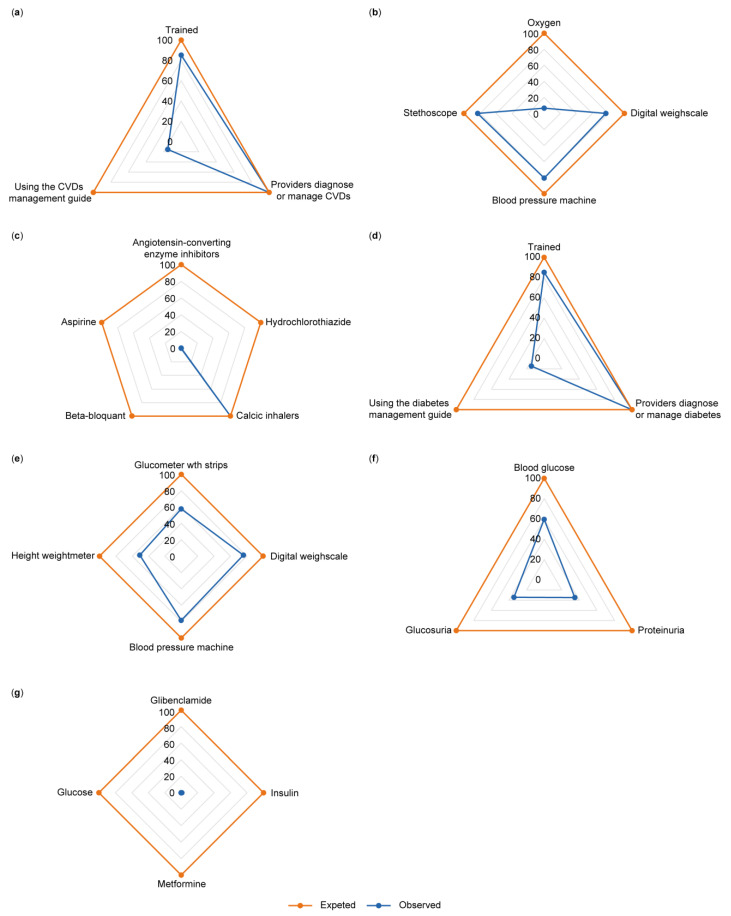
Descriptions of domains for assessing the level of readiness to manage CVD and diabetes. (**a**) Staff trained in diabetes management and the use of guidelines for CVD. (**b**) Technologies and the availability of basic functional equipment for CVD. (**c**) The availability of essential drugs to manage CVD. (**d**) Staff trained in diabetes management and the use of guidelines for diabetes. (**e**) Technologies and the availability of basic functional equipment for diabetes. (**f**) The ability to diagnose diabetes. (**g**) The availability of essential drugs to manage diabetes.

Figure 3 describes the domains for assessing readiness to manage CVD. As shown in Figure 3a, the training domain reported that all HFs have providers who can manage and diagnose diabetes, 85.2% of these HFs have staff trained in management and diagnosis, and only 15.3% of HFs use a guide to manage CVD. Concerning technologies and the availability of basic functional equipment (Figure 3b), 6.5% of HFs have oxygen devices, 76.8% have digital weighing scales, 80.6% have blood pressure machines, and 82.9% have stethoscopes. Concerning the availability of essential drugs to manage CVD (Figure 3c), 0.1% of HFs have angiotensin-converting enzyme inhibitors, 0.1% have hydrochlorothiazide, 100% have calcic inhalers, 0.1% have beta-blockers, and 0.1% have aspirin.

Figure 3 also describes the domains related to the assessment of readiness to manage diabetes. Concerning staff trained in diabetes management and the use of guidelines (Figure 3d), all HFs have providers who can manage and diagnose diabetes, 85.2% of HFs have staff trained in diabetes management and diagnosis, and only 14.5% of HFs use a diabetes management guide. Concerning technologies and the availability of basic functional equipment(Figure 3e), 57.1% of HFs have a glucometer with strips, 76.8% have a digital weighing scale, 80.6% have a blood pressure machine, and 51.1% have a height–weight meter. Concerning the ability to diagnose diabetes (Figure 3f), 59.6% of HFs can determine blood glucose, 34.8% can determine proteinuria, and 34.3% can determine glucosuria. Regarding the availability of essential drugs to manage diabetes (Figure 3g), 0.1% of HFs have glibenclamide, 0.8% have insulin, 0.1% have metformin, and 0.2% have glucose.

### 3.2. Level of Readiness of DRC HFs to Manage CVD and Diabetes (Score ≥ 70%)

**Figure 4:**
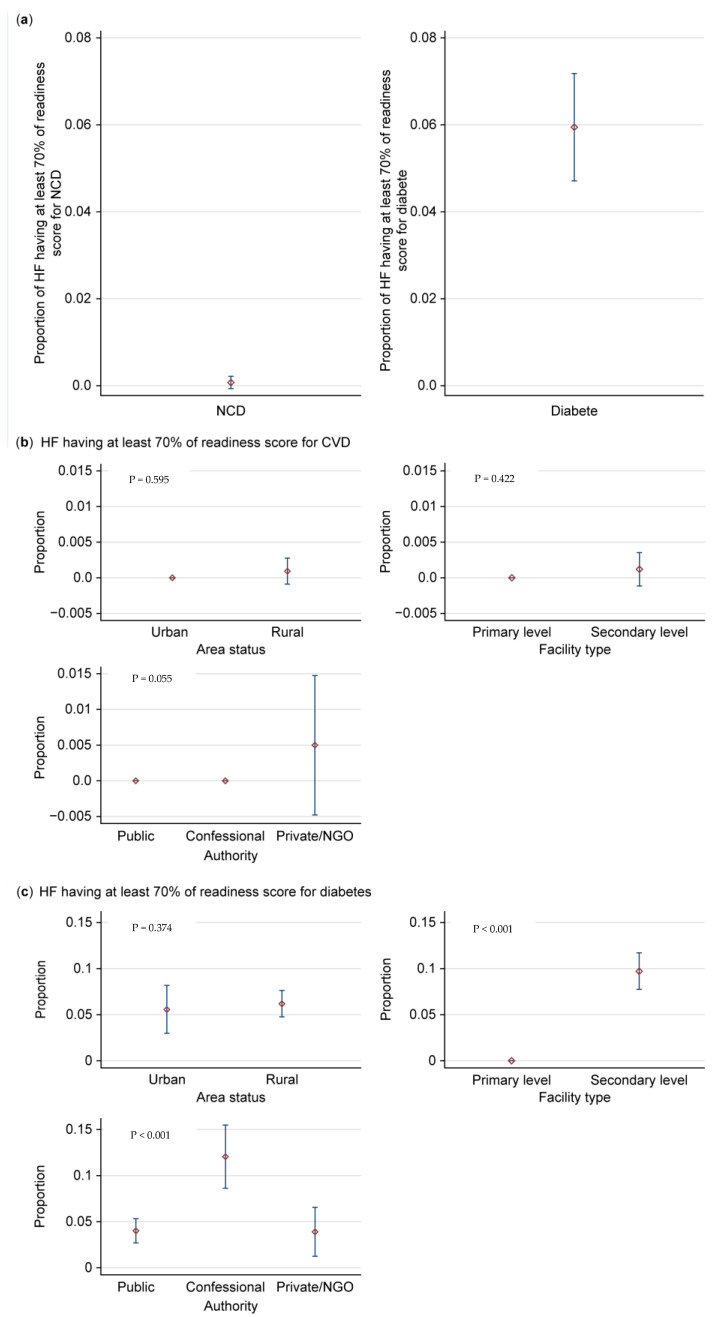
Level of readiness of DRC HFs to manage CVD and diabetes (score ≥ 70%) (HF: health facility). (**a**) Overall assessment. (**b**) Stratified assessment in relation to CVD. (**c**) Stratified assessment in relation to diabetes.

Overall, the analyses (Figure 4) reported that only 0.07% (CI95%: 0.01–0.5) of DRC HFs are prepared to manage CVD, and 5.9% (CI95%: 4.8–7.3) are prepared to manage diabetes (Figure 4a). When stratified for CVD (Figure 4b), rural HFs were non-significantly more prepared to manage CVD (1.25%) than urban HFs (*p* = 0.595). Secondary-level HFs were non-significantly more prepared to manage CVD (1.25%) than primary-level HFs (*p* = 0.422), and private/NGO HFs were non-significantly more prepared to manage CVD (5%) than public and denominational HFs (0.055). According to Figure 4c, rural HFs were also non-significantly more prepared to manage diabetes (6.3% (CI95%: 5.0–7.5)) than urban HFs (*p* = 0.374). Secondary HFs were significantly more prepared to manage diabetes (9.8% (CI95%: 7.5–12.0)) than primary HFs (*p* < 0.001), and denominational HFs were significantly more prepared to manage diabetes (12.0% (CI95%: 8.7–15.2)) than public and private/NGO HFs (*p* < 0.001). No province possesses over 50% of health facilities equipped to address cardiovascular illnesses. The provinces with health facilities exhibiting the least preparedness in managing cardiovascular illnesses and diabetes are Nord Ubangi and Sankuru.

### 3.3. Level of Readiness of HFs to Manage CVD and Diabetes in DRC Provinces

Estimated median scores by province are shown in Table 2. No province possesses over 50% of health facilities equipped to address cardiovascular illnesses. The provinces with the lowest median scores in managing CVD are Nord Ubangi and Sankuru (Table 2a). Only four provinces (Haut Uele, Kinshasa, Nord Kivu, and Sud Kivu) possess over 50% of health facilities equipped to address diabetes. The provinces with the lowest median scores in managing diabetes are Nord Ubangi and Sankuru (Table 2b).

Only 0.07% (CI95%: 0.01–0.5) of HFs had a score ≥70% for CVD management, and 5.9% (CI95%: 4.8–7.3) had this score for diabetes management (Figure 4).

## 4. Discussion

The DRC’s healthcare facilities were unprepared to address cardiovascular disease and diabetes. Merely 0.07% of HFs achieved a score ≥ 70% for cardiovascular disease management, while 5.9% attained this grade for diabetes control. In the stratified analysis, it was seen that despite the inadequate overall readiness, urban, secondary, and confessional HFs are more equipped to manage cardiovascular disease and diabetes than rural, primary, public, and private healthcare facilities. Physical accessibility to high-quality services that align with clients’ needs is a primary role of a health system. Healthcare institutions should possess the capability to deliver services, as suggested across many tiers, encompassing service readiness and service availability. As anticipated, secondary-level facilities exhibited superior readiness and availability relative to primary-level facilities across all evaluated areas. The study’s findings illustrate the prevalence of preparedness to manage CVD and diabetes. This confirms that health systems encounter substantial obstacles in the preparedness and capacity to address non-communicable diseases, including diabetes.

Insufficient preparedness to address cardiovascular disease and diabetes has also been documented in research conducted in other low- and middle-income nations [15,18]. The HFs in the DRC are unprepared to deliver comprehensive treatment services for cardiovascular disease and diabetes. Human resources are inadequately prepared, and fundamental equipment, laboratory supplies, and important management medications remain unavailable. These findings align with various studies that assessed HFs in low- and middle-income nations [18,21,22,23]. The observed low scores could be attributed to the government’s prioritization of communicable diseases (CDs) in its support for the DRC’s health system and technical and financial partners’ concentration on CD-related areas.

As in Ghana [24], the DRC’s healthcare system encompasses diverse levels of care and lacks convergent and uniform national documentation and texts on CVD and diabetes management. The outcomes of this study will allow political and administrative authorities to make choices and help sectoral technical experts to establish strategic and operational papers, as well as standards and recommendations, for CVD and diabetes. It has been noticed that in more than half of sub-Saharan African nations, the generation of normative papers, guidelines, or regulatory texts in support of the battle against CVD and diabetes is not based on scientific evidence [25].

The findings of this study should compel political and administrative authorities, along with the government’s technical and financial partners, to reevaluate their allocation and support strategies for the healthcare system. Considering the ongoing epidemiological change, a comprehensive approach to health system support is the sole effective and sustainable method to enhance the health system and mitigate morbidity and death burdens. It has been asserted that enhancing the health system is the most suitable response to the health and health system difficulties confronting any nation [24].

The outcomes of this study have substantial policy implications for CVD and diabetes care in the DRC. The strategic objective of the universal health coverage (UHC) goal is to strengthen primary-level institutions to enable them to offer preventive and promotive health services. Generally, the study’s findings suggest that primary-level facilities are less prepared to manage diabetes based on the CVD and diabetes service availability and preparedness categories assessed. UHC priority actions to ensure universal access to NCD services include (1) raising the number of persons receiving such treatment; (2) broadening the package of NCD services that are given; and (3) decreasing the cost of accessing these services. To address the disparity between population demands and healthcare requirements for diabetes and, by extension, non-communicable diseases (NCDs), health facilities must be furnished with the necessary equipment, medications, and personnel, presented as a comprehensive and integrated package for NCD management. Furthermore, public health promotion initiatives and education can mobilize a significant portion of the population to recognize that chronic disorders are as critical as acute diseases, are frequently lifelong, and are closely associated with lifestyle choices. These activities predispose individuals to the risk of non-communicable diseases and are strongly entrenched in society’s standards. Awareness campaigns should primarily focus on fostering a cultural shift in societal perceptions of health-related wellbeing and risky behaviors.

## 5. Strengths and Limits

To our knowledge, this study encompasses the highest number of HFs among those utilizing the SARA technique. This is also the first study conducted in the DRC to implement the SARA methodology. This study may exhibit information bias, as some responses were self-reported by the participants; for instance, no verification source was mandated to confirm staff training. The study’s strength resides in its consideration of HFs from all HDs.

Several limits must be recognized. A restriction is that the readiness indicators were evaluated using the WHO-SARA approach, which exclusively concentrated on supply-side factors, such as infrastructure, supplies, commodities, and human resources. This methodology would not have completely elucidated the dynamic interactions and specific elements that affect the wider health system components [26]. Moreover, certain data obtained comprised the respondents’ verbal replies, which could not be substantiated. Consequently, it may have led to bias. Finally, these data are from 2018 and may no longer accurately represent current reality; yet, they come from the sole source available addressing our study topic.

Considering the age of the data utilized in this study (about 7 years old), recent data are essential to assess the current service readiness in the DRC to handle CVD and diabetes, and to hence verify trends in the availability and readiness of the healthcare system to provide CVD and diabetes services in multiple political and insecurity crises context in comparison to the data gathered in 2018.

## 6. Conclusions

Significant deficiencies must be rectified to enhance service delivery in the management of cardiovascular disease (CVD) and diabetes. Most primary-level and rural facilities demonstrated inadequate preparedness in CVD and diabetes screening and management, exhibiting low readiness scores and limited-service availability in the assessed domains. While secondary-level services are relatively accessible, critical gaps persist that must be addressed to improve readiness for CVD and diabetes care. Healthcare facilities should possess the capacity to deliver the recommended services across various tiers, ensuring both service readiness and availability. This would facilitate early detection and the initiation of treatment, which is essential for preventing or delaying the onset of CVD, diabetes, and associated complications. In light of the observed results, the following five recommendations are proposed to the governing party and its technical and financial collaborators: (i) formulate standardized national protocols for the management of cardiovascular disease (CVD) and diabetes across various levels of healthcare facilities and establish mechanisms to ensure compliance; (ii) implement systems to guarantee that healthcare providers receive ongoing training and retraining to enhance their competencies in CVD and diabetes management; (iii) ensure that all healthcare levels are supplied with essential medications and appropriate diagnostic equipment for the management of CVD and diabetes; (iv) incorporate quality improvement initiatives, such as supervision and mentoring programs, to enhance observed outcomes; and (v) reevaluate health system resource allocation and support, prioritizing non-communicable diseases (NCDs) within the context of epidemiological transition.

## Figures and Tables

**Table 1:** Tracer elements in the respective domains for CVD and diabetes services.

Domains	Tracers
Diabetes	
Staff trained in diabetes management and use of guidelines	Existence of trained staff
	Providers to diagnose or manage diabetes
	Using the diabetes management guide
Technologies and availability of basic functional equipment	Glucometer wth strips
	Digital weighscale
	Blood pressure machine
	Height–weight meter
Ability to diagnose diabetes	Blood glucose
	Proteinuria
	Glucosuria
Availability of essential drugs to manage diabetes	Glibenclamide
	Insulin
	Metformine
	Glucose
Cardiovascular Diseases (CVDs)	
Staff trained in CVDs management and use of guidelines	Existence of trained staff
	Providers to diagnose or manage CVDs
	Using the diabetes management guide
Technologies and availability of basic functional equipment	Oxygene
	Digital weighscale
	Blood pressure machine
	Stethoscope
Availability of essential drugs to manage CVDs	Angiotensin-converting enzyme inhibitors
	Hydrochlorothiazide
	Metformine
	Calcic inhalers
	Beta-blockers
	Aspirin

**Table 2:** Level of readiness of HFs to manage CVD and diabetes in DRC provinces. (**a**) Level of readiness (%) of HFs to manage CVD in DRC provinces. (**b**) Level of readiness (%) of HFs to manage diabetes in DRC provinces.

(**a**)						
Province	N	Mean	SD	p25	Median	p75
Bas-uele	41	30.5	21.1	0.0	41.7	41.7
Equateur	41	40.4	8.7	41.7	41.7	41.7
Haut uele	44	40.0	8.3	41.7	41.7	41.7
Haut-katanga	72	45.5	10.9	41.7	41.7	50.0
Haut lomami	41	32.9	16.9	25.0	41.7	41.7
Ituri	73	35.0	19.5	25.0	41.7	41.7
Kasaï	53	35.7	12.4	33.3	41.7	41.7
Kasaï central	59	33.9	20.2	25.0	41.7	41.7
Kasaï oriental	44	40.7	9.7	41.7	41.7	41.7
Kinshasa	73	40.1	10.1	41.7	41.7	41.7
Kongo central	80	43.1	6.5	41.7	41.7	41.7
Kwango	43	34.7	18.0	25.0	41.7	41.7
Kwilu	70	41.0	7.3	41.7	41.7	41.7
Lomami	42	43.7	9.5	41.7	41.7	50.0
Lualaba	41	47.6	9.0	41.7	41.7	58.3
Mai-ndombe	50	40.3	12.9	41.7	41.7	41.7
Maniema	56	42.9	6.2	41.7	41.7	41.7
Mongala	41	38.6	15.7	41.7	41.7	41.7
Nord ubangi ‡	41	16.9	20.0	0.0	0.0	41.7
Nord-kivu	94	45.6	16.8	41.7	41.7	58.3
Sankuru ‡	41	20.3	24.0	0.0	0.0	41.7
Sud ubangi	41	44.7	9.1	41.7	41.7	41.7
Sud-kivu	62	42.2	19.0	41.7	41.7	58.3
Tanganika	42	21.6	19.7	0.0	20.8	41.7
Tshopo	54	36.1	16.1	33.3	41.7	41.7
Tshuapa	41	38.2	16.2	41.7	41.7	41.7
(**b**)						
Province	N	Mean	SD	p25	Median	p75
Bas-uele	41	27.6	22.7	0.0	31.3	45.8
Equateur	41	40.1	16.3	25.0	39.6	54.2
Haut uele	45	39.0	15.8	25.0	39.6	47.9
Haut-katanga	74	49.5	18.8	39.6	56.3	62.5
Haut lomami	41	32.3	21.0	18.8	31.3	45.8
Ituri	77	39.3	26.5	12.5	47.9	62.5
Kasaï	55	31.9	17.8	18.8	31.3	45.8
Kasaï central	59	36.6	24.6	16.7	39.6	56.3
Kasaï oriental	44	43.8	17.7	31.3	39.6	61.5
Kinshasa	73	49.7	13.5	39.6	56.3	58.3
Kongo central	80	48.3	12.3	39.6	45.8	57.3
Kwango	43	37.6	20.7	25.0	45.8	54.2
Kwilu	70	40.9	11.7	33.3	39.6	45.8
Lomami	42	40.8	16.3	31.3	41.7	54.2
Lualaba	41	49.0	16.6	31.3	45.8	62.5
Mai-ndombe	51	40.6	17.5	33.3	39.6	52.1
Maniema	60	40.5	16.3	31.3	42.7	49.0
Mongala	41	32.5	19.2	25.0	25.0	43.8
Nord ubangi §	41	19.0	23.7	0.0	0.0	39.6
Nord-kivu	98	53.5	21.6	45.8	62.5	68.8
Sankuru §	41	24.9	29.7	0.0	0.0	56.3
Sud ubangi	41	41.1	18.1	25.0	33.3	58.3
Sud-kivu	71	46.4	27.8	18.8	58.3	68.8
Tanganika	46	24.3	25.2	0.0	14.6	45.8
Tshopo	55	33.8	19.5	25.0	33.3	45.8
Tshuapa	41	33.7	19.8	18.8	31.3	45.8

SD: Standard deviation. ‡: The two provinces with the lowest median scores in managing cardiovascular illnesses. §: The two provinces with the lowest median scores in managing diabetes.

## Data Availability

Data are available online at https://dhsprogram.com/data/available-datasets.cfm (accessed on 15 May 2023).
